# Oral health status and treatment needs of children and young adults attending a day centre for individuals with special health care needs

**DOI:** 10.1186/1472-6831-8-30

**Published:** 2008-10-22

**Authors:** Folakemi A Oredugba, Yinka Akindayomi

**Affiliations:** 1 Department of Child Dental Health, College of Medicine, University of Lagos, PMB 12003, Idi-Araba, Lagos, Nigeria; 2 Children's Developmental Centre, Lagos, Nigeria

## Abstract

**Background:**

The oral health condition of individuals with special health care needs have been reported in literature to be influenced by various sociodemographic factors, including living conditions and severity of impairment. This study was carried out to determine the oral health status and treatment needs of children and young adults attending a day institution for those with special needs.

**Methods:**

This study was carried out as part of an oral health screening program organized by the institution and consent was obtained from parents and guardians before the screening. All information was supplied by the parents during the screening using a questionnaire completed by the dentist. Oral examination was carried out on all consenting subjects in attendance on the days of screening in the school clinic with parents and teachers in attendance, using standard World Health Organisation oral health indices to assess dental caries, oral hygiene status, malocclusion and other oral health parameters.

**Results:**

Fifty-four subjects aged 3–26 years (mean 12.28 ± 6.82 years) and comprising 72.2% males and 27.8% females participated in the study. Over 90% were from parents of high and middle level educational background. Thirty-six (66.7%) were caries free, with a mean dmft score of 0.7 ± 1.77 and mean DMFT score of 0.4 ± 1.44 with no significant difference across gender (p = 0.5) and parents' educational status (p = 0.43). The mean OHI-S of the total population in this study was 1.36 ± 0.16. Females had a mean score of 0.88 ± 1.10 while males had a mean score of 1.55 ± 1.24 with no significant difference (p = 0.6). Twenty-five (46.3%) had good oral hygiene, 17 (31.5%) had fair oral hygiene and 12 (22.2%) had poor oral hygiene, with no significant difference across gender (p = 1.11) and age groups (p = 0.07). Fifteen (27.8%) had gingivitis with no significant difference across age groups (p = 0.17). Forty-five (83.3%) had Angle's class I malocclusion, 6(11.1%) class II and 3 (5.6%) class III. Chronologic enamel hypoplasia was found in 9 (16.7%) of the total population. Up to 53.7% of the total population will require oral prophylaxis, 33.3% required restorations on their posterior teeth and 12.9% required veneers for labial facing of hypoplastic enamel.

**Conclusion:**

The subjects in this study had a high prevalence of dental caries and need for restorative care. They would benefit from parental education on diet modification, improvement of oral hygiene practices and regular dental visits.

## Background

Individuals with special health care needs have been reported in literature to have poorer oral hygiene and periodontal status, more untreated caries and fewer remaining teeth [1-3]. They are those who have physical, mental, sensory, behavioral, cognitive, emotional and chronic medical conditions which require health care beyond that considered routine, and which involves specialized knowledge, increased awareness, attention and accommodation [4].

Their oral health condition may be influenced by age, severity of impairment and living conditions. Individuals with special needs may have great limitations in oral hygiene performance due to their potential motor, sensory and intellectual disabilities [5-7], and so are prone to poor oral health. This group of individuals may also not understand and assume responsibility for or cooperate with preventive oral health practices [8]. Those who are very young, those with severe impairments, and those living in institutions are dependent on parents, siblings or caregivers for general care including oral hygiene. Many care givers do not have the requisite knowledge or values to recognize the importance of oral hygiene and do not themselves practice appropriate oral hygiene or choose a proper diet [9]. They may be more susceptible to dental caries if they reside at home and are pampered with cariogenic snacks and other unhealthy eating habits. Studies on select populations show that children with special health care needs have both more dental problems and more untreated dental disease relative to other children [10,11].

Poor oral health conditions have also been linked to low socio-economic status. Poor and nearly poor children with special health care needs and those with greater limitations attributable to disability were more likely to have unmet dental care needs [12]. Earlier studies on this group of individuals in our environment show that they had high unmet needs, especially periodontal treatment needs [13-15]. This study was carried out to determine the oral health status and treatment needs of those from a different socioeconomic background to those from previous studies in the same environment, using the same criteria, in order to provide information for future planning and intervention.

## Methods

The study population consisted of children and young adults attending a private day centre for individuals with special needs in an urban area of Lagos. A similar study was carried out earlier, using the same criteria and tools, on children and young adults attending three public schools for individuals with special needs in the same area in Lagos [15]. At the time of this study, there were ninety-five persons attending the private institution. The subjects were examined during an oral health screening session organized by the institution. Approval for this study was obtained from the Research, Grants and Ethics Experimentation Committee of the College of Medicine, University of Lagos, Nigeria. Consent forms were also sent to parents, to indicate their children's or wards' participation in the screening exercise. Only subjects whose parents consented to their ward's participation and were present in school at that time were examined. The screening for each subject included a record of the child's bio-data, type of disability and parents' educational background, as provided by the parent or from the school record. A parent's educational level was classified as 'high' when either parent had attended a tertiary institution, 'middle', when either parent had attended secondary school and 'low' when they had attended only primary school or no education [16].

One of the authors (FAO), carried out the oral examination on all the subjects in the school clinic using natural light. They were examined for the following parameters using the World Health Organization Oral Health Survey Basic Methods [17]:

### Dental caries

using the decayed, missing and filled teeth (dmft) index for primary (0–5 years) and early mixed dentition (6–19 years) and Decayed, Missing and Filled Teeth (DMFT) index for late mixed (11–15 years) and permanent dentitions (16 years and above). A tooth was considered decayed when there was frank carious cavitation on any surface of the tooth. A tooth was classified as missing in the index if it was extracted due to caries. A tooth was classified as filled if it had a restoration for a carious lesion. Exfoliated teeth in the primary and mixed dentition, unerupted and those extracted for other reasons apart from caries were not included in the indices.

### Oral hygiene status

using the Simplified Oral Hygiene Index (OHI-S) of Greene and Vermillon. The oral hygiene of each child was classified as 'good' when the OHI-S score was 0–0.9, 'fair' when it was 1.0–1.9 and 'poor' when it was 2.0 up to 6.

### Occlusion anomalies

Angle's classification of occlusion was used to classify malocclusion. Crowding, spacing and anterior open bite were also recorded.

### Chronologic enamel hypoplasia

Consistent discoloured malformations on teeth of the same series in at least two quadrants.

### Missing teeth

A tooth was classified as missing if it had not erupted after six months of its expected eruption date.

### Retained teeth

A tooth was classified as retained if it was still in the arch after six months of its expected date of exfoliation.

Fracture of anterior teeth was also recorded.

Findings were communicated to the parents/guardians, appropriate oral health education given and written referrals given to the dental clinic where necessary.

### Data analysis

Data obtained were analyzed with the health statistical software Epi info version 6 [18]. Chi square test, t-test, Kruskal-Wallis test, Bartlett's test and Fisher's exact test were used where applicable when comparing findings across age groups, gender and parents' educational background.

## Results

Sixty-six (69.5%) subjects responded to the call for screening. Twelve were either ill or very uncooperative so were excluded from the study, leaving 54 (56.8%) subjects, aged 3–26 years (mean age 12.28 ± 6.82 years) who were examined. There were 37 (72.2%) males and 15 (27.8%) females. Thirty five (64.8%) were from parents of high educational level, 18 (33.3%) were from the middle and only 1 (1.9%) was from the low educational level and so grouped with the middle educational level. There were 13 (24%) subjects in each of the 0–5 and 6–10 years age groups, 10 (18.6%) in the 11–15 years age group and 9 (16.7%) each in the 16–20 and 21 ≤ years age groups (Table 1). One (1.8%) subject had attention deficit hyper activity disorder (ADHD), 11 (20.4%) autism, 21 (38.9%) cerebral palsy, 5(9.3%) Down syndrome, 14 (25.9%) learning disability and 2 (3.7%) seizure disorder.

**Table 1:** Socio-demographic characteristics of the study population

**Characteristics**	**N**	**(%)**
**Gender**		

Male	39	(72.2)

Female	15	(27.8)

**Age (years)**		

Range	3–26 years	

0–5	13	(24.0)

6–10	13	(24.0)

11–15	10	(18.6)

16–20	9	(16.7)

20<	9	(16.7)

**Parents' Educational Level**		

High	35	(64.8)

Middle	19	(35.2)

**Total**	**54**	**(100.0)**

Thirty-six (66.7%) were caries free. The mean dmft of the primary and early mixed dentition years was 0.7 ± 1.77 while the mean DMFT of the late mixed dentition and permanent dentition was 0.4 ± 1.44 as shown in Table 2. The mean dmft/DMFT for females and males was 0.66 ± 1.17 and 1.3 ± 2.41 respectively with no significant sex difference (Kruskal-Wallis test for two groups = 0.41; p = 0.5) (Table 3). The mean dmft/DMFT of subjects of parents with high educational level was 1.14 ± 2.37 while that of those from middle level was 1.11 ± 2.02, with no significant difference (p = 0.43).

**Table 2:** Mean dmft/DMFT of the study population according to age group

**Age group (years)**	**dmft**	**DMFT**
<5	1.46 ± 2.06	-

6–10	1.46 ± 2.69	-

11–15	-	0.5 ± 1.26

16–20	-	0.77 ± 1.98

21<	-	1.11 ± 2.61

Total mean dmft = 0.7 ± 1.77		

Total mean DMFT = 0.4 ± 1.44		

**Table 3:** Mean dmft/DMFT according to gender

**Gender**	**Obs**	**Total carious teeth**	**Mean**	**Maximum**
**Female**	15	10	0.66 ± 1.17	3

**Male**	39	51	1.30 ± 2.41	8

Kruskal-Wallis test = 0.41				

p = 0.5				

The mean OHI-S of the total population in this study was 1.36 ± 0.16. Females had a mean score of 0.88 ± 1.10 while males had 1.55 ± 1.24 with no significant sex difference (Bartlett's test = 0.26; p = 0.6).

**Figure 1:**
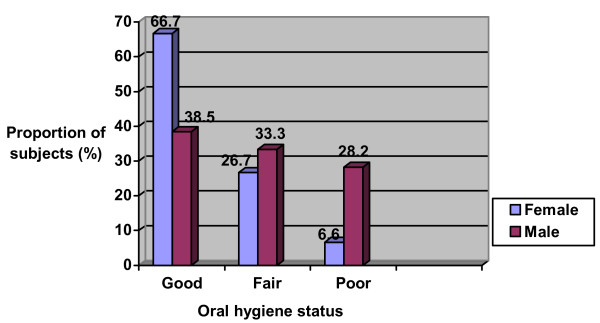
Oral hygiene status of the study population according to gender.

**Figure 2:**
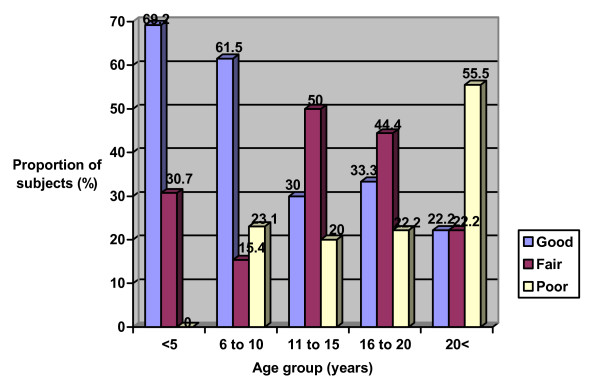
Oral hygiene status of the study population according to age group.

**Figure 3:**
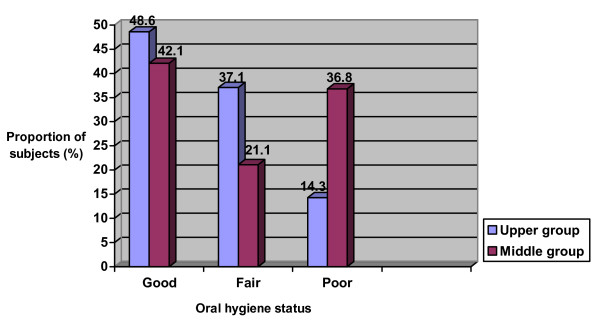
Oral hygiene status according to parents' educational status.

Twenty-five (46.3%) had good oral hygiene, 17 (31.5%) had fair oral hygiene and 12 (22.2%) had poor oral hygiene, with no significant difference across sex (p = 1.11), age groups (p = 0.07) and parents' educational level (p = 0.17) (Figures 1, 2 and 3).

**Figure 4:**
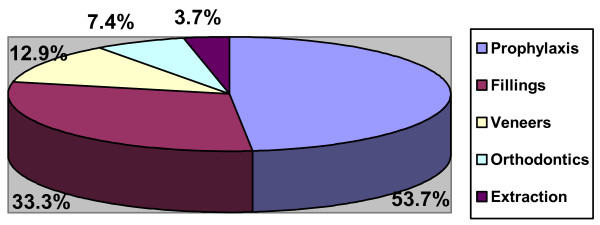
Treatment needs of the study population.

Fifteen (27.8%) had gingivitis with no significant difference across gender (Fisher's exact test = 0.05, p = 0.59), parents' educational level (Chi sq = 2.61, p = 0.26) and age groups (Chi sq = 6.3, p = 0.17). Gingivitis was significantly present in subjects with seizure disorders, learning disability, Down syndrome and autism (Chi sq = 14.95, p = 0.01). Forty-five (83.3%) had Angle's class I malocclusion, 6(11.1%) class II and 3 (5.6%) class III. Five (24%) of those with cerebral palsy had class II malocclusion while 3 (60%) of those with Down syndrome had class III malocclusion. Chronologic enamel hypoplasia was found in 9 (16.7%) of the total population, with no significant difference across the different disabilities (Chi sq = 3.84, p = 0.57) and age groups (Chi sq = 4.40, p = 0.35). Anterior open bite was seen in 9.3%, crowding in 12.9%, fractured anterior teeth (3.7%) and retained teeth (3.7%) with no significant difference across age group, gender and parents' education level. Up to 53.7% of the total population will require oral prophylaxis, 33.3% required restorations on their posterior teeth and 12.9% required veneers for labial facing of hypoplastic enamel (Figure 4).

## Discussion

The institution where this study was carried out is a private institution, therefore patronized mostly by parents from the upper and middle socioeconomic status. Twenty-four percent had attended the dental clinic for treatment previously. This finding shows a better exposure to oral health care services than those subjects from public schools (3.6%) seen in an earlier study from the same environment and of comparable age range [15]. It is expected that the higher the educational level of an individual, the better the health seeking behaviour of that individual and the family members.

The majority of the subjects were caries-free, although the proportion of caries-free subjects was relatively low compared with that of subjects with special needs in public schools (93%). Some authors have however reported a lower caries prevalence in children with disabilities compared with those without disabilities [19,20]. The conflicting results from different studies are due to different age groups, severity of impairments and type of residence of the population studied. The major component of the 'decayed, missing and filled teeth' index was the decayed teeth (dt) which is similar to findings from studies in other countries [21,22]. Some of the reasons given for increased occurrence of dental caries in this group of individuals are increased thirst, 'eating for consolation' or 'comfort' consumption of sweets and drinks [23] and long-term consumption of medications in form of sweetened syrups. When parents attend the clinic with their children, it is important they are educated on the need to reduce and as much as possible to substitute cariogenic snacks with fruits and vegetables.

Only two children have had an amalgam and Glass Ionomer Cement restorations. Although this proportion who had received dental care is indeed small, it is encouraging because previous surveys in this environment showed no index of restorative care in the large population studied [15].

In contrast to dental caries, almost half of the subjects in this study (46.3%) had good oral hygiene compared with lower proportions of those in earlier studies in this environment and elsewhere but among children from parents of lower educational background [14,15,24]. This shows that the educational status of parents has a positive effect on the dental care of persons with SHCN [25]. These individuals require help for oral hygiene performance irrespective of their medical condition in order to achieve good oral cleanliness. There was also no significant difference in the oral hygiene status between females and males, and age groups in this study. This is because most of the subjects are dependent on parents or care givers to carry out their routine oral hygiene activities. These findings confirm earlier reports that the prevalence of dental disease tends to be affected by demographic factors [26].

A high prevalence of unmet needs is still evident in this study despite the educational background of the parents and the fact that the school and residence of the subjects are located in an urban area of the state. Other studies in developed countries have shown that dental care is the most prevalent unmet health care need for children with special health care needs [1-3,27]. There are various factors which create barriers to receiving oral health care even among the elite; these include low priority placed on oral health by parents and chronicity of oral diseases. If oral health is not perceived as being important, the children would not be taken for dental check-up [28]. A family's inability to be committed to the children's dental care may also result from lack of understanding of the long-term health risks that may burden a child who does not receive urgently-needed care [29]. In these individuals, oral health needs are competing with already burdensome chronic health conditions. The consequences of unmet oral health care needs include infection of the oral tissues, negative behaviour and aggravation of concomitant medical conditions [30-32]. This group of children would also not be able to complain when in pain so the condition may go un-noticed until it reaches the acute phase. The children may also not cooperate in the dental chair. In this case other forms of behaviour management methods may be utilized by the attending dentist for effective delivery of care.

More than half of the subjects with Down syndrome (DS) had class III malocclusion which is similar to reports from previous studies on those with DS [33]. Class II malocclusion was also more prevalent in those subjects with cerebral palsy. A few participants in this study (7.4%) would benefit from orthodontic treatment with the support of parents. Orthodontic treatment had been carried out successfully in some patients with disabilities [34]. Intellectual or physical impairments should not be a barrier to receiving orthodontic care. Rather, the dentist should critically assess the severity of the malocclusion, the possible effects of leaving the case untreated as well as establish realistic goals and outcomes of treatment [35]. For such patients, greater reliance may have to be placed on care givers for the maintenance of satisfactory oral hygiene [36] which is required for successful orthodontic treatment.

Primary health care providers may influence access to dental care by oral health assessment and prompt dental referral [12,37]. One of the current themes in disability policy is the promotion of partnership with all key stakeholders including people with disabilities and their families and carers [38], such as this screening exercise. The establishment of relationships with family support groups to reach parents and other caregivers will improve the oral health of the children [39]. This study is limited by the small number of subjects who participated in the screening. Some subjects did not return their consent forms, some were not in school on the days of screening, some were very ill and some were uncooperative so excluded from participating. All these factors are common in such institutions. The enrolment in such institutions are however increasing and more institutions are being established so larger populations are expected in future studies.

## Conclusion

There was a high prevalence of dental caries and need for restorative care among the subjects in this study. Their oral hygiene was better than that of subjects attending public schools from previous studies. It is recommended that regular contact be made with parents and caregivers and educated on the need for diet modification, improvement in oral hygiene and regular dental visits for their wards.

## Competing interests

The authors declare that they have no competing interests.

## Authors' contributions

FAO conceived of the idea, drafted the questionnaire, carried out the oral examination, analyzed the data and took part in the draft of the manuscript. YO participated in the draft of the manuscript.

## Pre-publication history

The pre-publication history for this paper can be accessed here:


